# *Translational Neurodegeneration* in the era of fast growing international brain research

**DOI:** 10.1186/s40035-021-00276-9

**Published:** 2022-01-02

**Authors:** Jialin C. Zheng, Shengdi Chen

**Affiliations:** 1 Translational Neurodegeneration, Departments of Neurology and Regenerative Medicine, Tongji Hospital, Tongji University School of Medicine, Shanghai, China; 2 Translational Neurodegeneration, Department of Neurology & Institute of Neurology, Rui Jin Hospital, Shanghai Jiao Tong University School of Medicine, Shanghai, China

Another year has flown by and we have entered 2022. The challenges in 2021 have continued to occur as we unceasingly fight against the pandemic. 2021 will be remembered for the world’s perseverance and dedication to overcome extremely adverse circumstances. At the beginning of 2022, we are celebrating the 10th birthday of our journal, *Translational Neurodegeneration* (TN). Inaugurated in 2011 under the editorship and leadership of Dr. Shengdi Chen, our mission is to promote and foster rapid conversion of basic science research to clinical applications by offering a high-visibility forum for groundbreaking translational research in neurodegenerative diseases and to ultimately benefit not only patients but also societies.

During the past ten years, we have been witnessing splendid progresses in TN. While we received only 31 submissions in 2012, in 2021 by Nov. 22nd the number of submissions has rocketed to 274. TN received her first impact factor in 2017 (5.872) and it reached 8.014 in 2020. The number of BMC and Springer yearly downloads of TN publications was around 26,000 in 2012. During the following ten years, the yearly downloads was increased by more than ten-folds, reaching an estimated 350,000 in 2021.

While we are facing historical challenges of COVID-19, we are also facing significant challenges of a fast-growing aging society. According to a report released by the United Nations, 1 in 11 world population was over 65 in 2019 and by 2050 the ratio will be almost doubled to 1 in 6. As the global population ages, the prevalence of neurodegenerative disorders, including Alzheimer’s disease (AD) and Parkinson’s disease (PD), is fast increasing. Notably, the number of cases of dementia in the developed world is projected to rise from 13.5 million in 2000 to 21.2 million in 2025, and to 36.7 million in 2050. The number of deaths caused by AD is now on par with the number of deaths caused by stroke, which is the third most common leading cause of death in the world. Unfortunately, we still do not fully understand the pathogenesis of AD and other neurodegenerative disorders; we still lack reliable early diagnosis markers, let alone effective therapies for these diseases. What makes the matter worse is in the case of AD that these patients are usually diagnosed only after symptoms appear, that is 10–20 years after the initial pathological changes of the brain, thus making it almost impossible to reverse or slow down the disease progression. For PD, the second most common neurodegenerative disease after AD, we are facing these daunting challenges as well.

With the rapid advance in big data, genomics, proteomics, single cell analysis, and even single extracellular vesicle technology, we believe that a new horizon has just emerged in the fields of translational neurodegeneration. These new technologies along with multidisciplinary approaches will undoubtedly open new frontiers in discovering early diagnosis methods and developing treatment for these diseases. For example, scientists are trying to find AD biomarkers for early diagnosis using optical coherence tomography (OCT), a noninvasive technology that examines retina microcirculation.

The newly launched China Brain Initiative and also the effort of international collaboration in international brain initiatives will help to accelerate our progress. Unmasking neurodegenerative disease pathogenesis and identifying innovative diagnostics or therapeutics are the common goals of mainstream brain initiatives around the world. Through cooperation we will be able to learn cutting-edge methods/technologies and to adjust plans accordingly and timely.

As our societies continue to age, neurodegenerative diseases are increasingly placing a tremendous social and economic burden on both the patients and the communities. Finding causes, treatments and cures for these diseases are crucial. In *TN*, we will strive to do our utmost best to facilitate research to achieve these goals in the next ten years. We have identified the following critical areas for translational neurodegenerative diseases research: (1) To identify and validate biomarkers for early or pre-clinical diagnosis and for use as endpoints of clinical progression through strategies such as proteomics, genomics, metabolomics and functional imaging techniques in combination of artificial intelligence; (2) To promote clinical development through the latest innovative technologies such as neuroimaging strategies, stem cell technology, exosome technology and nanotechnology; and (3) To accelerate drug development and marketing through big cohort clinical trials; and more importantly, these clinical efforts will generate a multitude of new ideas and contacts that can be fed back for more focused/targeted research. Thus, the mission of our journal in translational neurodegenerative diseases is to, and will continue to, bridge critical basic research with important clinical applications.

We would like to encourage more scientists, especially young scientists, in different subjects and research areas to collaborate and to dedicate to translational neurodegenerative diseases. Our collective goal is to elucidate the pathogenesis, to establish innovative diagnosis, to develop novel interventions, to find out cures for these diseases, and to enlighten the future of patients and the whole society. *Translational Neurodegeneration* will continue to serve as a high-visibility forum to foster and expedite the rapid conversion of basic science and clinical research to benefit patients. The concept and spirit of translational research in the field of neurodegenerative disorders will spread through our *journal* and through our authors and readers.

We are extremely grateful to the authors, the referees, the editors and associate editors. We also thank Springer Nature, our publisher, for their help in making this fast-growing journal possible in the past ten years. Special thanks also go to Drs. Jianqing Ding and Yuyan Tan, managing editors of *Translational Neurodegeneration*, to the leaders, colleagues, team members of Rui Jin Hospital. In addition, we are indebted to the leaders of Shanghai Jiao Tong University, the Neurodegenerative Disorders Division of Chinese Neuroscience Society, the Parkinson’s Disease & Movement Disorder Division of Chinese Medical Association, whose help is instrumental for the success of this undertaking.

## Data Availability

not applicable.

